# An early-life challenge: becoming an older sibling in wild mandrills

**DOI:** 10.1098/rsos.240597

**Published:** 2024-07-24

**Authors:** Axelle Delaunay, Océane Cossu-Doye, Berta Roura-Torres, Loïc Sauvadet, Barthélémy Ngoubangoye, Elise Huchard, Marie J. E. Charpentier

**Affiliations:** ^1^ Institute of Evolutionary Biology of Montpellier (ISEM), Université de Montpellier, CNRS, IRD, EPHE, Montpellier, France; ^2^ Behavioral Ecology and Sociobiology Unit, German Primate Center, Leibniz Institute of Primate Research, Göttingen, Germany; ^3^ Department of Sociobiology/Anthropology, Institute of Zoology and Anthropology, Johann-Friedrich-Blumenback, Georg-August University Göttingen, Göttingen, Germany; ^4^ Projet Mandrillus, SODEPAL, Bakoumba, Gabon; ^5^ Centre International de Recherches Médicales de Franceville, Franceville, Gabon; ^6^ Department for the Ecology of Animal Societies, Max Planck Institute of Animal Behavior, Bücklestrasse 5, Konstanz 78467, Germany

**Keywords:** mother–offspring conflict, mother–offspring relationship, sibling birth, sibling rivalry, transition to siblinghood, weaning

## Abstract

In monotocous mammals, most individuals experience the birth of a younger sibling. This period may induce losses in maternal care and can be physiologically, energetically and emotionally challenging for the older sibling, yet has rarely been studied in wild primates. We used behavioural data collected from a natural population of mandrills to investigate changes in maternal care and mother–juvenile relationship throughout the transition to siblinghood (TTS), by comparing juveniles who recently experienced the birth of a younger sibling, to juveniles who did not. We found that the TTS was associated with an abrupt cessation of the weaning process for the juvenile, and to a decrease in maternal affiliation. Juveniles’ reactions were sex-specific, as males associated less with their mother, while females tended to groom their mother more often after the birth of their sibling. Despite the substantial loss of maternal care, juveniles did not show an increase in conflict or anxiety-related behaviours. This study contributes to explain why short interbirth intervals often pose a risk to juveniles’ survival in monotocous primates. Our results contrast existing studies and further highlight the importance of examining the TTS in species and populations with various life histories and ecologies.

## 1. Introduction

Parental investment is defined as any form of care provided to an offspring that increases the offspring’s fitness at the cost of the parent’s future reproduction [1]. The amount of parental investment that can be devoted to each offspring being limited, several conflicts are expected to arise between the different family members. On the one hand, parents and offspring should conflict over the quantity of parental investment, with offspring trying to get more investment than the parents are willing to provide, and on the other hand, competition should arise between offspring, who should all try to monopolize parental investment at the expense of their siblings [2,3].

Sibling competition to access and monopolize parental care [2] has been extensively studied across taxa (for reviews, see [4,5]). Sibling competition can impact offspring’s development, with long-term consequences on their morphology (e.g. insects [6]; birds [7]; mammals [8,9]), physiology (e.g. birds [10–12]; mammals [13–15]) and behaviour (e.g. birds [16,17]; mammals [14,18]), and can ultimately impact their fitness, for instance in species where siblicide is common (e.g. birds [19–21]; mammals [9,22]). Most empirical studies to date have, however, focused on brood- or litter-rearing species (especially in mammals; for reviews, see [5,23,24]), where same-age siblings compete over the same maternal resources at the same time. Most of the theoretical models and evolutionary hypotheses have thus been developed for intrabrood competition [24]. By contrast, interbrood competition, where siblings are not at the same developmental stage [5], has been largely neglected so far, which is surprising given that Trivers’ original model was developed for species producing singletons [2].

Monotocous mammals, which produce one offspring at a time, offer unique opportunities to study interbrood competition between individuals who may not depend on the same maternal resources [24]. These species generally have long developmental periods, often with a juvenile phase, during which offspring develop strong and enduring bonds with their mother that can extend way beyond infancy (e.g. yellow baboons, *Papio cynocephalus* [25,26]; Asian elephants, *Elephas maximus* [27]; killer whales, *Orcinus orca* [28]; red deer, *Cervus elaphus* [29]). Weaned offspring keep benefitting from post-weaning forms of maternal care, such as protection against predators, facilitated access to food or social support [30]. Several empirical studies have recently suggested that these post-weaning maternal resources could improve offspring’s growth (e.g. chimpanzees, *Pan troglodytes* [31]), future reproductive success, and longevity (chimpanzees 32,33]; red deer [34]). Monopolizing such maternal resources—if they can be monopolized—could therefore be advantageous for offspring, meaning that sibling competition could last long beyond lactation or nutritional dependency.

In line with this, recent empirical studies showed that sharing maternal resources between different-age siblings could be costly for offspring. In Galapagos fur seals (*Arctocephalus galapagoensis*) and sea lions (*Zalophus wollebaeki*) for instance, mothers can still be nursing their previous offspring by the time they give birth to the next one, and the older sibling may attempt to prevent the newborn from nursing or directly attack it, reducing its survival probability by 50% [35]. Similarly in false vampire bats (*Megaderma lyra*), Leippert *et al*. [36] reported a case of a newborn being killed by its older, weaned sibling. In several primate species, including in humans, short interbirth-intervals (IBIs) can reduce survival for both the older (in rhesus macaques, *Macaca mulatta* [37]; in yellow baboons [38]; in humans, *Homo sapiens* [39–41]) and the younger sibling [37], and can even have intergenerational effects [42]. Despite such evidence, behavioural studies in monotocous mammals have thus far focused much more on mother–offspring conflict around weaning (for review, see [43]), where maternal milk is the main resource at stake, than during later developmental milestones, such as the birth of a younger sibling, or ‘transition to siblinghood’.

In primates, previous studies reported contrasting changes in mother–juvenile relationships throughout the transition to siblinghood. In captive and free-ranging cercopithecines, the birth of a younger sibling induces an abrupt decrease in time spent in close spatial proximity and in the interaction rate between the mother and the juvenile, often driven by the juvenile itself [44–47]. Maternal aggression and rejection increases, often accompanied by signs of stress and ‘depression’ in the offspring [44–46,48], such as tantrums (a behaviour commonly considered as a behavioural manifestation of mother–offspring conflict [43,49]) or self-directed behaviours (generally indicating anxiety [50–52]). In wild chacma baboons (*Papio ursinus*), maternal behaviour does not change following the birth of a new infant but juveniles nonetheless solicit their mother more often and show more signs of distress (especially those that experience this transition at an older age) than juveniles without a sibling [53]. In wild bonobos (*Pan paniscus*), juveniles face an abrupt fivefold increase in cortisol level after the birth of their sibling, which endures for more than six months, although they show little behavioural changes [54]. In industrialized modern human societies, the arrival of a younger sibling is often characterized by a decrease in maternal care and in the rate of mother–child interactions, with a switch in who initiates interactions—from mother to children becoming the primary initiator [55–58]. Confrontational behaviours with the mother increase, as well as signs of distress and clinging/demanding behaviours in the older sibling—especially in children experiencing this transition at a younger age [55,58,59]. Children’s behavioural adjustment shows high interindividual variability in humans [55,58], however, and its quality (i.e. relatively positive versus negative reactions to the birth of the younger sibling) is associated, potentially causally, with the quality of siblings’ relationship later in life [60,61].

Importantly, species life-history traits might influence juveniles’ adjustment to the transition to siblinghood. For instance, the birth of a younger sibling, when it triggers weaning or co-occurs with it, might induce a harsher transition than in species where IBIs are longer. In line with this, juvenile rhesus macaques can nurse up to 1 day before the birth of their sibling, following which they experience an abrupt cessation of nursing, and those who experience steeper decreases in nursing and maternal care show more signs of distress in the first month after the birth of their sibling [44,62]. On the contrary, in chacma baboons, juveniles have already been weaned several months before their mother gives birth, and the birth is not associated with any major change in mother–offspring relationship [53]. Therefore, these two developmental milestones, weaning and transition to siblinghood, do not always occur simultaneously across species or individuals. For instance, in some populations, including mandrills, high-ranking females can conceive when they are still lactating, while subordinate females display a slower reproductive pace [63], which could translate into a harsher transition to siblinghood for offspring born to higher-ranking females. In addition, juveniles’ adjustment to the birth of a younger sibling could also be influenced by genetic relatedness between siblings, as predicted by the kin selection theory [2,64]. For instance, in species characterized by a high-reproductive skew and a long male tenure length, like in mountain gorillas (*Gorilla beringei beringei*), full siblings may be common, which could induce a smoother transition (but see [65] for the effect of relatedness on siblings’ relationships). In contrast, in species with lower relatedness between siblings, as in mandrills, where males’ reproductive skew is high but tenure length is relatively short [66], juveniles could experience a harsher transition after the birth of their sibling.

In this study, we investigated changes affecting mother–juvenile relationships following the birth of a younger sibling in a long-lived social primate, the mandrill (*Mandrillus sphinx*). Mandrills live in huge multi-male–multi-female groups, sometimes up to several hundred individuals, mainly composed of females and their offspring [67]. Mandrills form matrilineal societies [68], where females are philopatric and inherit their rank from their mother, while males disperse upon sexual maturity. Mandrills are seasonal breeders, with most births occurring during the rainy season [63], meaning that most offspring experience the birth of their younger sibling either during the next birth season, or two seasons later, so that IBIs often present a bimodal distribution. Females give birth to one offspring [69], which they nurse for eight months on average in captivity [70]. Importantly, female mandrills can be lactating and pregnant simultaneously [63], which means that the birth of a younger sibling may accelerate or terminate abruptly the weaning process, enhancing competition among siblings.

Here, we investigated the transition to siblinghood (hereafter, TTS) in the only natural population of habituated mandrills, using behavioural data collected from 2015 to 2022. We hypothesized that TTS would be associated with a loss of maternal care (H1), a weaker mother–offspring bond (H2), a greater role of juveniles (relatively to mothers) in maintaining the mother–offspring bond (H3) and an increase in juveniles’ anxiety-related behaviours (H4). Following a recent study in chacma baboons [53], we compared mother–juvenile interactions among juveniles with no younger sibling and those of similar age who recently experienced the birth of a younger sibling, and tested the following predictions: (P1a) following the birth of a younger sibling, juveniles would no longer be nursed or carried by their mother, independently of their age and sex, while juveniles without a younger sibling would show a more gradual age-related decline for these two behaviours; (P1b) juveniles who underwent TTS at a young age would show a more abrupt decrease in nursing and maternal carrying than juveniles who experienced the birth of a younger sibling at an older age. In addition, (P2) grooming interactions and spatial association between the mother and the juvenile would decrease, and (P3) juveniles would initiate a greater proportion of grooming interactions, and be more proactive at maintaining spatial proximity to their mother following the birth of their younger sibling. Finally, (P4) juveniles who recently experienced the birth of a younger sibling, and especially those who experienced it at a young age, would display more tantrums and self-directed behaviours than those who did not. For (P2−P4), we explored potential sex-differences in juveniles’ reactions to TTS, as sex-differences in mother–offspring bond and social behaviour typically emerge early during development in cercopithecines [71–73].

## 2. Material and methods

### 2.1. Study site and population

We studied a natural population of habituated mandrills who freely range in the Lékédi Park and its surroundings, located in southern Gabon, daily monitored since 2012 by the Mandrillus Project. This population originated from 65 individuals initially living in a semifree-ranging population housed at Centre International de Recherches Médicales de Franceville, Gabon, who were released in two waves in the Lékédi Park, in 2002 and 2006 [74]. Captive-born females started to breed with wild males in 2003, and in late 2022, only four females out of 250 individuals in the group were captive-born. All individuals, including infants and juveniles, are recognizable by trained observers using facial and body features. During daily monitoring, observers on foot record data on individual life-history, developmental trajectory and behaviour. In this study, we used data collected on 191 juveniles between January 2015 and October 2022.

### 2.2. Demographic data

Individual birth dates were assessed with certainty when observed in the field (*n* = 88 infants), or estimated within a time-window of 1 day to two months for the remaining 103 infants, based on patterns of mother’s sexual swellings and infants’ physical appearance. Individual birth order was inferred based on maternal reproductive history when known. We then divided birth order in three different classes—first-, second- and later-born (birth order greater than or equal to three)—as juveniles’ reaction to TTS may differ with maternal experience.

### 2.3. Behavioural data

Since 2015, trained observers collected daily ad libitum and 5 min focal behavioural observations on all individually recognized infants and juveniles [75]. During focal observations, we recorded the occurrence of suckling, ventral carrying and grooming bouts with the mother. For grooming bouts, we recorded the direction of the interaction, and the identity of the individual who initiated the grooming event. Mandrills typically initiate grooming either by starting to groom their partners (when they are the groomer) or by approaching them and standing in a grooming position (e.g. head down, limbs outstretched, presenting the body part they want to be groomed). We also collected the occurrence of tantrums and anxiety-related behaviours (self-scratch and yawning) displayed by the focal individuals. Tantrums are often considered a behavioural manifestation of mother–offspring conflict [43,49], and are characterized in mandrills by a set of distinctive vocalizations such as moaning, gecking and screaming [49]. Maintenance of spatial proximity was assessed by recording every close approach or leave (to and from 1 m) between focal individuals and their mother. Finally, we collected scans every 2 min during focal observations, during which we recorded the identity of all groupmates—including the mother—that were in body contact and within 5 m around the focal individual.

Mothers’ social rank was established using the outcomes of approach–avoidance interactions during ad libitum and focal samplings performed on adult females. We computed yearly linear hierarchies using normalized David’s score. Individuals’ social ranks were highly correlated between years [63], therefore, we assigned each female a unique relative rank from 2012 to 2022. We then divided adult females into three classes of rank of similar size (high-, medium- and low-ranking [76].

In this study, we aimed to investigate the short-term behavioural response to the birth of a younger sibling. Following Delaunay *et al*. [53], we compared juveniles who recently experienced the birth of a younger sibling—within the three months following the birth of the newborn—with juveniles who did not yet. In our sample of focal observations, juveniles who had experienced the birth of a younger sibling were 11−32 months old. Therefore, we restricted our dataset to this age range. For juveniles without a younger sibling, we included any focal observations and scans collected on juveniles aged 11−32 month old and until the birth of their younger sibling or until they stopped being followed (when older than 32 months). For juveniles with a younger sibling, we included any focal observations and scans collected during the first three months following the birth of their sibling. Data from juveniles who experienced the birth of a younger sibling more than three months ago were excluded from the study, as their TTS was considered as over. When a newborn died before three months of age, we excluded all observations of the older sibling following this death. Individuals who were observed both before and after the birth of their younger sibling were included in both groups, respectively (with and without a younger sibling, *n* = 72). We used a total of 4866 focal observations (mean ± s.d. = 25.48 ± 28.66 per individual) and 6989 scans (mean ± s.d. = 36.40 ± 41.88 per individual) from 191 individuals (*n* = 79 with a younger sibling, mean age ± s.d. = 21.1 ± 4.6 months; *n* = 184 without a younger sibling, mean age ± s.d. = 16.3 ± 3.6 months) born to 76 mothers.

### 2.4. Statistical analyses

#### 2.4.1. TTS effects on maternal care

To characterize the impact of TTS on maternal care, we used two measures: suckling frequency and ventral carrying frequency. For each focal observation, we recorded whether the juvenile: (i) suckled or (ii) was carried ventrally by its mother at least once (yes = 1, no = 0). Preliminary inspection of the data revealed that juveniles with a younger sibling were no longer suckling or being carried by their mother, so we provided below a descriptive analysis.

#### 2.4.2. TTS effects on maternal grooming

To assess the effect of TTS on maternal grooming, we first recorded, for each focal observation, whether the focal juvenile received grooming from its mother at least once (binary, yes/no—model 1), and whether it groomed its mother (binary, yes/no—model 2). We modelled the probability to receive or give grooming to the mother with two generalized linear mixed models (GLMMs) with a binomial error structure.

Second, to investigate the effect of TTS on juveniles’ role in initiating grooming interactions with the mother, we considered every grooming event for which the initiator was known, and recorded whether it was initiated by the juvenile (1) or by its mother (0) (binary, initiated by the juvenile yes/no—model 3). We restricted this dataset to the first grooming event recorded during a focal observation, and discarded any other grooming event that occurred after this first event, regardless of the direction, because we considered these events as potentially non-independent. We modelled the probability that a grooming bout was initiated by the juvenile (versus the mother) using a GLMM with a binomial error structure (model 3) on 396 grooming bouts with a known initiator.

#### 2.4.3. TTS effects on mother–juvenile spatial proximity

To investigate mother–juvenile proximity, we first used scan data to estimate how often a juvenile was found in close proximity to the mother. For each scan observation, we recorded whether the juvenile focal was found in proximity (1) or not (0) to its mother. We considered two distinct ranges of proximity: body contact (binary, yes/no—model 4), and within 5 m from the mother (binary, yes/no—model 5). We ran two GLMMs with a binomial error structure.

Second, we used focal data to characterize juveniles’ role in the maintenance of spatial proximity. We considered every approach and leave between the mother and the focal juvenile, and recorded whether it was initiated by the juvenile (1) or by the mother (0). Using 1199 approaches and 1350 leaves between the mother and the focal juvenile, we ran two GLMMs with a binomial error structure to investigate the probability that an approach or a leave were initiated by the juvenile (binary, yes/no—models 6 and 7, respectively).

#### 2.4.4. TTS effects on mother–juvenile conflict

To investigate the effect of TTS on mother–juvenile conflict, we considered two measures: juvenile tantrums and juvenile anxiety-related behaviours (yawning and self-scratches). For each focal observation, we recorded whether: (i) a tantrum occurred (binary, 1/0—model 8) and (ii) the juvenile displayed at least one anxiety-related behaviour (binary, 1/0—model 9). We ran two GLMMs with a binomial error structure.

#### 2.4.5. Fixed and random effects

For each model, we tested the effect of having recently experienced the birth of a younger sibling (yes/no), and included the following fixed effects: focal juvenile’s age (in months), sex, birth order and maternal rank. We tested the interaction terms between the recent birth of a younger sibling and the focal juvenile’s age and sex (except for model 8 because tantrums were rarely recorded). We also included two additional fixed effects: (i) in model 3, grooming direction (i.e. grooming received versus given) and its interaction term with the birth of a younger sibling to test whether juveniles with a younger sibling initiate more often grooming interactions with their mother than juveniles without a younger sibling, and especially initiate more often the grooming events where they are groomed by their mother; (ii) in most models, duration of the focal observation to account for time out-of-view (in seconds) (except in models 3, 6 and 7 because the probability that a behaviour is initiated by the juvenile did not depend on the duration of the observation, and models 4 and 5 because based on scan data). In all models, we included the year of observation as a random effect, and the focal juvenile’s identity nested in the mother identity to account for repeated observations. The structure of each model (fixed effects and random effects), along with sample sizes and corresponding predictions are summarized in electronic supplementary material, table S1.

#### 2.4.6. Statistical methods

All statistical analyses were performed using the R Studio software (v. 4.0.2). We ran mixed models using the ‘*glmmTMB*’ function from the glmmTMB package [77], and the ‘*bglmer*’ function from the blme package [78] to confirm the results with a Bayesian approach whenever we obtained a singular fit. We assessed fit singularity using the function ‘*check_singularity*’ from the performance package [79]. To control for the age of focal juveniles, we first visually checked the relationship between age and our response variables. Whenever this relationship was not linear, we fitted univariate models with a linear, a second- or third-degree polynomial function to model the effect of age (random effects were also included) and compared model fits, selecting the model with the lowest Akaike information criterion (AIC) [80]. All quantitative variables were *z*-transformed (mean = 0, s.d. = 1) using the ‘*scale*’ function from the car package [81] to facilitate model convergence and to compare effect sizes across estimates [82]. To diagnose the presence of multi-collinearities, we computed the variance inflation factor (VIF) for each predictor of each model using the function ‘*check_collinearity*’ from the performance package. VIFs were inferior to 2 in all cases, indicating that multi-collinearities did not impact coefficients’ estimation in our models. To test the significance of our fixed factors, we calculated the likelihood ratio test (LRT) and its associated *p*-values for each model using the ‘*drop1*’ function, and computed the 95% Wald confidence intervals. Non-significant interactions were removed from the full model to limit the risk of overparametrization and facilitate the interpretation of simple effects. Finally, we checked the distribution of the residuals using ‘simulateResiduals’ from the DHARMa package [83].

## 3. Results

### 3.1. TTS effects on maternal care

**Figure 1.**
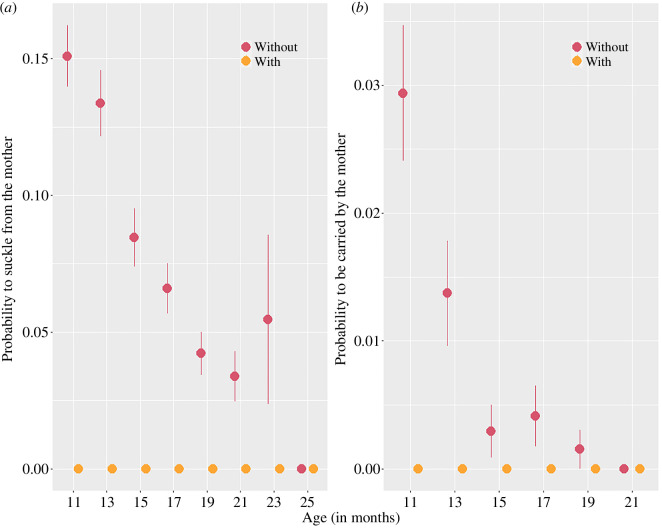
Probability to (*a*) suckle and (*b*) be carried by the mother during a focal observation according to juvenile’s age and sibling status, using raw data from 4866 focal observations on 191 juveniles. In all panels, “With” refers to juveniles who recently experienced the birth of a younger sibling and “Without” those who did not. For graphical purposes, we pooled values per age class so that “11” represent the mean probability to suckle/be carried for all juveniles aged 11–12 months old, “13”, juveniles 13 and 14 months old etc. In (*a*), “25” represents the pooled values for all juveniles older than 25 months old as suckling was no longer observed after this age, and in (*b*), “21” represents the pooled values for all juveniles older than 21 months old as carrying was no longer observed after this age. Dots represent the average raw value for a given age class and vertical bars represent the standard errors.

Juveniles with a younger sibling were no longer nursed or carried by their mother, regardless of their age at the time of their younger sibling’s birth (figure 1*a,b*). In contrast, suckling and carrying probabilities gradually decreased with age for juveniles without a younger sibling and ceased around 25 months old for suckling (figure 1*a*) and 21 months old for carrying (figure 1*b*). Juveniles without a younger sibling were still suckling in approximately 15% of the observations at 11–12 months old (mean probability ± s.d. = 0.151 ± 0.360, i.e. once every 30 min), in approximately 7% at 17–18 months old (mean ± s.d. = 0.066 ± 0.248, i.e. once every 75 min) and in approximately 3% at 21–22 months old (mean ± s.d. = 0.034 ± 0.181, i.e. once every 145 min). Similarly, they were still being carried in approximately 3% of the observations at 11–12 months old (mean ± s.d. = 0.029 ± 0.169, i.e. once every 170 min), and in 0.4% at 17–18 months old (mean ± s.d. = 0.004 ± 0.064, i.e. once every 1250 min).

### 3.2. TTS effects on maternal grooming

**Figure 2.**
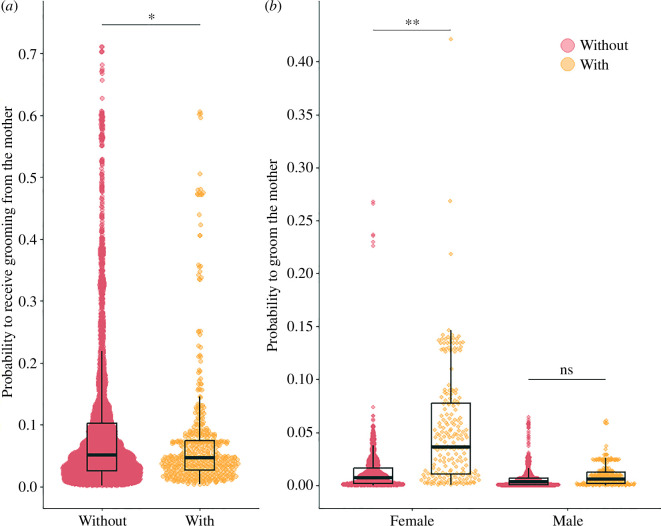
Influence of the birth of the younger sibling on mother–juvenile grooming interactions. In all panels, ‘With’ refers to juveniles who recently experienced the birth of a younger sibling and ‘Without’ those who did not. (*a*) Predicted probability to be groomed by the mother depending on the sibling status during a focal observation. (*b*) Predicted probability to groom the mother depending on the sibling status and focal juvenile’s sex (females on the left side of the graph, males on the right). Dots are fitted values from the models (obtained with the function ‘fitted’ from the package stats), and boxplots show the median of the distribution of the fitted values (black horizontal bar), the 25th and 75th quartiles (bottom and top of the boxes, respectively) and the whiskers include a maximum of 1.5 times the interquartile range. The effect of the predictor ‘Sibling status’ and the associated *p*-values are shown. ‘ns’: not significant (*p* > 0.05), *: *p* < 0.05, **: *p* < 0.01, ***: *p* < 0.001.

Juveniles in TTS were significantly less likely to be groomed by their mother than juveniles without a younger sibling (odds ratio (OR) = 0.58, mean probability ± s.d. = 0.08 ± 0.27 for juveniles with a younger sibling; 0.09 ± 0.29 for those without, table 1, figure 2*a*), although the difference in raw grooming probability was small between the two categories of juveniles. The probability to groom the mother tended to be influenced by the interaction between the sex of the focal juvenile and its sibling status (*p* = 0.091): female juveniles with a younger sibling tended to be more likely to groom their mother than females without a younger sibling (mean probability ± s.d. = 0.06 ± 0.24 for females with a younger sibling; 0.01 ± 0.11 for females without), while male juveniles tended to be equally likely to groom their mother independently of their sibling status (males with a younger sibling: 0.01 ± 0.10; without: 0.01 ± 0.08, table 1, figure 2*b*). Juveniles born to medium- or low-ranking females were also significantly less likely to be groomed by their mother than those born to high-ranking females (OR = 0.53 and 0.49, respectively, table 1), but maternal rank did not influence the probability to groom the mother. Juveniles’ age and birth order did not influence the probability to receive or give grooming to the mother, and both sexes were equally likely to be groomed by the mother (table 1).

**Table 1.** Results of the mixed models analysing the probability to receive (model 1) and give (model 2) grooming to the mother during a focal observation. Estimates, 95% confidence intervals (CIs), LRT statistics and *p*-values of the predictors were estimated using 4866 focal observations on 191 juveniles (*n* = 79 with a younger sibling, *n* = 184 without). Juvenile identity nested in mother identity, and year of observation were included as random effects. For categorical predictors, the reference category is indicated between parentheses. Significant effects are indicated in bold.

fixed factor	level	estimate	2.5%	97.5%	LRT	*p*-value
model 1: probability to receive grooming from the mother						
intercept		−1.511	−2.278	−0.745	—	—
presence of a younger sibling (no)	yes	**−0.546**	**−1.006**	**−0.086**	**5.641**	**0.018**
juvenile’s age		0.096	−0.045	0.237	1.777	0.182
juvenile’s sex (female)	male	−0.249	−0.549	0.051	2.695	0.101
juvenile’s birth order (first-born)	second-born	−0.410	−0.894	0.073	3.632	0.163
	later-born	−0.019	−0.381	0.343		
maternal rank (high-ranking)	medium-ranking	**−0.627**	**−1.021**	**−0.233**	**15.292**	**<0.001**
	low-ranking	**−0.720**	**−1.088**	**−0.353**		
focal duration		**0.708**	**0.555**	**0.861**	**105.755**	**<0.001**
model 2: probability to groom the mother						
intercept		−4.595	−5.778	−3.411	—	—
presence of a younger sibling (no)	yes	1.379	0.337	2.421	—	—
juvenile’s age		0.157	−0.183	0.497	0.710	0.400
juvenile’s sex (female)	male	−0.904	−1.744	−0.064	—	—
juvenile’s birth order (first-born)	second-born	−0.774	−1.923	0.376	3.014	0.222
	later-born	−0.669	−1.561	0.223		
maternal rank (high-ranking)	medium-ranking	0.133	−0.844	1.110	0.763	0.683
	low-ranking	−0.266	−1.249	0.717		
focal duration		**1.317**	**0.691**	**1.942**	**33.206**	**<0.001**
presence of a younger sibling (no)*Juvenile’s sex (female)	(yes, male)	−1.337	−3.015	0.340	2.849	0.091

Juveniles with and without a younger sibling were as likely to be the initiator of a grooming bout with their mother (table 2). Juveniles were significantly more likely to be the ones initiating grooming bouts with their mother as they grew older (OR = 1.47, table 2). Juveniles’ sex, birth order or maternal rank did not influence juveniles’ probability to be the ones initiating a grooming with the mother (table 2).

**Table 2.** Results of the mixed model testing the probability that a grooming event is initiated by the juvenile (versus the mother, model 3). Estimates, 95% CIs, LRT statistics and *p*-values of the predictors were estimated using 396 grooming events on 128 juveniles (*n* = 23 with a younger sibling, *n* = 120 without). Grooming direction refers to grooming received from the mother (mother–juvenile, *n* = 366) or given to the mother (juvenile–mother, *n* = 30). Juvenile identity nested in mother identity, and year of observation were included as random effects. For categorical predictors, the reference category is indicated between parentheses. Significant effects are indicated in bold.

fixed factor	level	estimate	2.5%	97.5%	LRT	*p*-value
model 3: probability that a grooming event is initiated by the juvenile						
intercept		1.340	−0.214	2.894	—	—
presence of a younger sibling (no)	yes	0.769	−0.270	1.808	2.371	0.124
juvenile’s age		**0.386**	**0.058**	**0.714**	**5.371**	**0.021**
juvenile’s sex (female)	male	−0.456	−1.224	0.313	1.626	0.202
grooming direction (juvenile–mother)	mother–juvenile	**−3.662**	**−4.980**	**−2.344**	**47.152**	**<0.001**
juvenile’s birth order (first-born)	second-born	0.092	−1.237	1.421	1.388	0.500
	later-born	0.494	−0.472	1.461		
maternal rank (high-ranking)	medium-ranking	0.533	−0.487	1.553	1.271	0.530
	low-ranking	0.157	−0.777	1.092		

### 3.3. TTS effects on mother–juvenile spatial proximity

**Figure 3.**
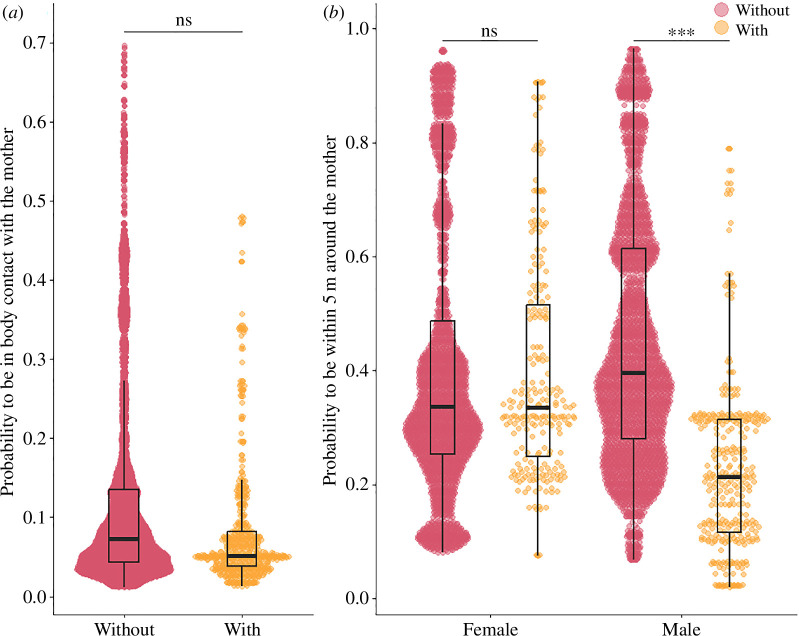
Influence of the birth of the younger sibling on mother–juvenile proximity. In all panels, ‘With’ refers to juveniles who recently experienced the birth of a younger sibling and ‘Without’ those who did not. (*a*) Predicted probability to be in body contact with the mother depending on the sibling status during a scan observation. (*b*) Predicted probability to be within 5 m around the mother depending on the sibling status and focal juvenile’s sex (females on the left side of the graph, males on the right). Dots are fitted values from the models (obtained with the function ‘fitted’ from the package stats), and boxplots show the median of the distribution of the fitted values (black horizontal bar), the 25th and 75th quartiles (bottom and top of the boxes, respectively) and the whiskers include a maximum of 1.5 times the interquartile range. The effect of the predictor ‘Sibling status’ and the associated *p*-values are shown. ‘ns’: not significant (*p *> 0.05), *: *p *< 0.05, **: *p* < 0.01, ***: *p* < 0.001.

Mother–juvenile proximity was partially influenced by the birth of a younger sibling: although the probability to be in body contact with the mother was similar in both groups (table 3, figure 3*a*), the probability to be within 5 m around the mother was significantly influenced by the interaction between juveniles’ sex and sibling status (table 3, figure 3*b*). Male juveniles with a younger sibling were less likely to be within 5 m of their mother than males without a younger sibling (mean probability ± s.e. = 0.23 ± 0.042 and 0.46 ± 0.50, respectively), while female juveniles were as likely to be within 5 m as those without (0.40 ± 0.49 for those with a sibling, 0.41 ± 0.49 for those without). Juveniles were less likely to be in body contact and within 5 m around their mother as they grew older (OR = 0.80 and 0.77, respectively), and first-born juveniles were more likely to be in body contact than later-born juveniles (OR = 1.67, table 3). Maternal rank did not predict the probability to be in body contact or within 5 m around the mother (table 3).

**Table 3.** Results of the mixed models analysing juveniles’ probability to be in body contact (model 4) or within 5 m around their mother (model 5) during a scan observation. Estimates, 95% CIs, LRT statistics and *p*-values of the predictors were estimated using 6989 scans on 192 juveniles (*n* = 78 with a younger sibling, *n* = 184 without). Juvenile identity nested in mother identity, and year of observation were included as random effects. For categorical predictors, the reference category is indicated between parentheses. Significant effects are indicated in bold.

fixed factor	level	estimate	2.5%	97.5%	LRT	*p*-value
model 4: probability to be in body contact with the mother during a scan observation						
intercept		−1.024	−1.732	−0.316	—	—
presence of a younger sibling (no)	yes	−0.287	−0.689	0.115	1.991	0.158
juvenile’s age		**−0.228**	**−0.340**	**−0.116**	**16.237**	**<0.001**
juvenile’s sex (female)	male	−0.147	−0.464	0.169	0.831	0.362
juvenile’s birth order (first-born)	second-born	**−0.619**	**−1.114**	**−0.124**	**6.123**	**0.047**
	later-born	**−0.328**	**−0.736**	**0.080**		
maternal rank (high-ranking)	medium-ranking	−0.196	−0.673	0.281	4.528	0.104
	low-ranking	−0.510	−0.974	−0.046		
model 5: probability to be within 5 m around the mother during a scan observation						
intercept		0.561	−0.214	1.337	—	—
presence of a younger sibling (no)	yes	0.107	−0.303	0.517	—	—
juvenile’s age		**−0.258**	**−0.339**	**−0.177**	**39.442**	**<0.001**
juvenile’s sex (female)	male	0.053	−0.293	0.400	—	—
juvenile’s birth order (first-born)	second-born	−0.061	−0.586	0.464	2.710	0.258
	later-born	−0.329	−0.767	0.109		
maternal rank (high-ranking)	medium-ranking	−0.015	−0.483	0.453	0.005	0.998
	low-ranking	−0.003	−0.461	0.456		
presence of a younger sibling (no)*juvenile’s sex (female)	(yes, male)	**−1.011**	**−1.543**	**−0.479**	**13.931**	**<0.001**

Juveniles were generally responsible for initiating an approach to their mother, independently from their sibling status (table 4). The probability to leave one another was similar for juveniles and their mother in general, and independent from the birth of a younger sibling (table 4). Juveniles born to higher-ranking females were more likely to initiate an approach to their mother (OR = 2.10, table 4). Juveniles’ age, sex and birth order did not influence the probability to approach or leave their mother, and the probability to leave the mother was independent from maternal rank (table 4).

**Table 4.** Results of the mixed models analysing the probability that an approach (model 6) or a leave (model 7) are initiated by the juvenile (versus its mother). Estimates, 95% CIs, LRT statistics and *p*-values of the predictors were estimated using respectively, 1199 approaches between the mother and the juvenile on 134 juveniles (*n* = 21 with a younger sibling, *n* = 128 without), and 1350 leaves on 141 juveniles (*n* = 33 with a younger sibling, *n* = 132 without). Juvenile identity nested in mother identity, and year of observation were included as random effects. For categorical predictors, the reference category is indicated between parentheses. Significant effects are indicated in bold.

fixed factor	level	estimate	2.5%	97.5%	LRT	*p*-value
model 6: probability that an approach is initiated by the juvenile						
intercept		2.645	1.764	3.526	—	—
presence of a younger sibling (no)	yes	−0.351	−1.206	0.503	0.758	0.384
juvenile’s age		−0.116	−0.278	0.045	1.900	0.168
juvenile’s sex (female)	male	−0.084	−0.400	0.231	0.144	0.705
juvenile’s birth order (first-born)	second-born	−0.263	−0.730	0.204	1.372	0.504
	later-born	−0.108	−0.505	0.289		
maternal rank (high-ranking)	medium-ranking	**−0.375**	**−0.874**	**0.125**	**12.111**	**0.002**
	low-ranking	**−0.742**	**−1.213**	**−0.270**		
model 7: probability that a leave is initiated by the juvenile						
intercept		−0.326	−0.910	0.257	—	—
presence of a younger sibling (no)	yes	0.201	−0.500	0.901	0.373	0.542
juvenile’s age		0.052	−0.086	0.189	0.400	0.527
juvenile’s sex (female)	male	−0.193	−0.471	0.085	1.920	0.166
juvenile’s birth order (first-born)	second-born	0.047	−0.379	0.473	2.640	0.267
	later-born	0.256	−0.101	0.614		
maternal rank (high-ranking)	medium-ranking	−0.212	−0.625	0.201	3.220	0.200
	low-ranking	0.094	−0.298	0.486		

### 3.4. TTS effects on mother–offspring conflicts

Tantrums were uncommon, and juveniles’ probability to throw a tantrum was independent from their sibling status, decreased as they grew older, and was lower for juveniles born to low-ranking females than those born to high- and medium-ranking females (OR = 0.39 and 0.53, respectively, table 5). Juveniles’ sex and birth order did not influence the probability to display a tantrum (table 5).

**Table 5.** Results of the mixed models analysing the probability to display a tantrum (model 8) and anxiety-related behaviours (model 9) during a focal observation. Estimates, 95% CIs, LRT statistics and *p*-values of the predictors were estimated using 4866 focal observations on 191 juveniles (*n* = 79 with a younger sibling, *n* = 184 without). Juvenile identity nested in mother identity, and year of observation were included as random effects. For categorical predictors, the reference category is indicated between parentheses. Significant effects are indicated in bold.

fixed factor	level	estimate	2.5%	97.5%	LRT	*p*-value
model 8: probability of tantrum						
intercept		−4.109	−5.098	−3.119	—	—
presence of a younger sibling (no)	yes	−0.373	−1.531	0.784	0.500	0.480
juvenile’s age		**−0.325**	**−0.609**	**−0.041**	**5.384**	**0.020**
juvenile’s sex (female)	male	0.489	−0.082	1.060	2.680	0.102
juvenile’s birth order (first-born)	second-born	−0.197	−1.049	0.655	0.749	0.688
	later-born	−0.299	−0.995	0.397		
maternal rank (high-ranking)	medium-ranking	−0.383	−1.089	0.322	**8.330**	**0.016**
	low-ranking	−1.024	−1.754	−0.293		
focal duration		**0.445**	**0.199**	**0.692**	**13.946**	**<0.001**
model 9: probability of anxiety-related behaviours						
intercept		0.202	−0.281	0.684	—	—
presence of a younger sibling (no)	yes	0.112	−0.161	0.385	0.700	0.403
juvenile’s age		−0.073	−0.154	0.008	3.280	0.070
juvenile’s sex (female)	male	0.061	−0.124	0.246	0.450	0.502
juvenile’s birth order (first-born)	second-born	−0.095	−0.384	0.194	2.006	0.367
	later-born	−0.164	−0.398	0.071		
maternal rank (high-ranking)	medium-ranking	**−0.027**	**−0.277**	**0.222**	**7.097**	**0.029**
	low-ranking	**0.247**	**0.009**	**0.484**		
focal duration		**0.669**	**0.603**	**0.734**	**440.446**	**<0.001**

Finally, juveniles with a younger sibling were as likely to display anxiety-related behaviours as juveniles without a younger sibling (table 5). Juveniles born to low-ranking females displayed significantly more anxiety-related behaviours than juveniles born to high- or medium-ranking females (OR = 1.28 and 1.32, respectively, table 5). Juveniles’ probability to display anxiety-related behaviours was independent from their sex, age and birth order (table 5).

## 4. Discussion

In this study, we investigated the behavioural changes in maternal care and mother–juvenile relationship during the TTS in wild mandrills. First, following our prediction (P1), we found that nursing and maternal carrying abruptly stopped after the birth of a younger sibling (P1a), thus juveniles who entered TTS at a younger age experienced a greater loss of maternal care (P1b). Second, we showed that juveniles with a younger sibling received less maternal grooming (P2), although the magnitude of this loss was small (they lost on average 1/10th of time of maternal grooming compared to their counterparts). Contrary to our prediction (P2), female juveniles with a younger sibling tended to groom their mother more often than females without a sibling (six times more), while males tended to groom their mother independently of their sibling status, and TTS did not impact juveniles’ probability to be the ones initiating a grooming bout with the mother in both sexes (P3). Third, we found that juvenile males spent less time close to their mother following the birth of their sibling, partially supporting our prediction (P2), although spatial proximity remained stable for juvenile females. However, juveniles from both sexes were mostly responsible for maintaining spatial proximity with their mother, independently from the birth of their sibling (contrary to P3). Finally, we showed that juveniles with a younger sibling neither displayed more tantrums nor signs of anxiety than those without (contrary to P4). We discuss below the implications of our findings for the understanding of parent–offspring conflict and sibling rivalry in primates.

First, mandrill mothers dramatically decreased their level of maternal investment toward their juvenile when giving birth, as they stopped nursing and carrying them, and groomed them less often, regardless of the age of their juvenile offspring. Thus, in mandrills, the birth of the younger sibling terminates the weaning process for the older sibling, as mothers are unable to nurse two offspring at the same time. In free-ranging, provisioned rhesus macaques, juveniles also stopped suckling their mother after the birth of their sibling [44], although mothers in this population can sometimes nurse two consecutive offspring at the same time [37]. By contrast, in chacma baboons and bonobos, juveniles were already weaned several months before their mother gave birth [53,54]. Mothers’ ability to nurse while being pregnant might largely depend on their ecology and life history. For instance, mandrills live in a rich environment where food is unlikely to be limiting, which, like provisioned macaques, might enable them to face the cumulated costs of late lactation and early pregnancy at the same time [63]. In contrast, chacma baboons live in an arid environment where food is drastically limited, which might force females to space out births by weaning their previous offspring before starting a new reproductive cycle. In bonobos and orang-utans, where the older juvenile is also weaned before the birth of the younger sibling, mothers may start a new reproductive cycle only when the older juvenile has reached nutritional independence and learnt most skills needed to extract food [54,84].

In addition to these two obvious forms of maternal care, mandrill mothers also groomed their juvenile less often, and this decrease did not seem to be driven by the mother or the juvenile in particular, as juveniles’ and mothers’ probability to be the one initiating grooming bouts or close proximity did not change with the TTS. These results partially resemble previous findings in macaques and industrialized human societies, where maternal grooming or maternal attention and responsiveness (in macaques and humans, respectively) decreased after the birth of a younger sibling [44,56,59]. However, juveniles’ reactions to these changes seem highly variable across studies and species: in the Cayo Santiago macaques, juveniles seemed to be responsible for the decrease in maternal grooming [44]. Maintaining a strong bond with the mother may be less critical in such free-ranging, provisioned population, where energy and mortality costs are reduced (no predator, supplemental feeding). In contrast, in humans, children initiated more interactions with their mother following the birth of a sibling, as a likely attempt to compensate for the loss of maternal attention [55–58]. In chacma baboons, although maternal behaviour did not change with TTS, juveniles nevertheless solicited their mother more often [53].

Surprisingly, age did not mediate the effect of TTS on mother–juvenile relationships. As the birth of the younger sibling terminated the weaning process for the juvenile and the loss incurred in terms of suckling was larger for young juveniles, we were expecting juveniles who entered TTS at a younger age to experience greater changes in their relationship with the mother. Indeed, in rhesus macaques, juveniles who experienced a greater decrease in nursing after their sibling’s birth showed more signs of distress [62]. In humans, as well, the increase in anxiety, clinginess and confrontational behaviours toward the mother after the birth of the newborn was more pronounced in younger children [55,59,85]. Recent studies in macaques and baboons also showed that having a too close in age younger sibling increased mortality risks at all ages for the older sibling [37,38], most likely because it induced a decrease in maternal investment. In mandrills, however, the birth of a younger sibling was perceived similarly and independently of age (and thus, of weaning). A potential explanation could be that juvenile mandrills adjust to their mother’s reproductive pace. Juveniles experiencing TTS at a younger age could speed up in the acquisition of autonomy when their mother becomes pregnant, so that they are ‘ready’ and independent enough for the birth of their younger sibling. This could explain why they do not express more anxiety-related behaviours nor initiate a larger proportion of the interactions with their mother when experiencing TTS earlier in life.

In contrast to two recent studies in wild baboons and bonobos [53,54], juvenile mandrills’ reactions to TTS appeared to be sex-specific. Indeed, juvenile males associated less often with their mother, while juvenile females tended to increase their grooming rate towards them. These differences may be triggered by several, non-mutually exclusive factors. First, the tendency of females to increase grooming could result from an attraction to the newborn, which is a common behaviour in primates, where juvenile and adult females often try to touch or handle infants of others [86–88]. A common way to gain access to the infant is to initiate a grooming with the mother [89–91]. Second, mandrills’ sex-specific reactions might reflect life history differences between sexes [72]. Indeed, in such matrilineal society, maintaining and/or reinforcing close social bonds with the mother could be more advantageous for juvenile females than for males, as strong social bonds with close maternal kin in adulthood translate into fitness advantages in female cercopithecines [92,93]. In these species, immature males typically form weaker bonds with their mother and are less integrated in their matriline, and such sex differences may emerge as early as the first year of life (e.g. [71–73,94]). Hence, the birth of a younger sibling may foster the independence of juvenile males, as in free-ranging rhesus macaques [44], while juvenile females may instead react by strengthening their bond with their mother. More generally, TTS may mark a turning point where juveniles start to balance the benefits of remaining attached with the mother (e.g. decreased predation risk, access to social learning and foraging skills) with the benefits of associating with peers (e.g. development of their own social network outside of their mother’s), which could lead to different trade-offs between sexes.

Despite facing a drastic decrease in maternal care, juvenile mandrills showed little indication that this transition was a source of conflict with their mother, or of anxiety. Indeed, they did not throw more tantrums or display more signs of anxiety, nor did they initiate a greater proportion of the interactions with their mother. Tantrums have often been considered as a conspicuous form of behavioural conflict over parental investment in primates, expressed during developmental milestones where mother and juvenile’s evolutionary interests diverge, such as weaning [43]. In mandrills, tantrums are often less conspicuous than in baboons (personal observation), and are already rare by the age juveniles enter TTS. Although the youngest juveniles were still able to throw tantrums, conflicts with the mother could be expressed through more subtle behaviours. In chacma baboons for instance, juveniles solicited their mother more often after the birth of their younger sibling, which suggests a conflict over the amount of maternal investment received [53]. Surprisingly, juvenile mandrills did not seem more anxious after the birth of their sibling. Studies in several cercopithecine species reported increased signs of distress or ‘depression’ during TTS [44,47,53,62]. In bonobos, a recent study reported a strong increase in cortisol level following the birth of a younger sibling, but they did not investigate whether this physiological change was associated with observable distress behaviours [54]. In mandrills, the birth of a younger sibling could also induce physiological changes that are not associated with observable distress behaviour. Alternatively, the lack of conflict and anxiety behaviours in mandrills could also reflect the absence of overt conflict with the mother, which could be the case if juveniles are already independent enough by the time their mother gives birth.

Finally, the decrease in maternal care induced by the birth of the younger sibling could translate into sibling rivalry [2]. As mothers can nurse and be pregnant at the same time in mandrills, pregnancy could represent a perfect case study of interbrood sibling competition, where the monopolization of maternal care through suckling by the older sibling could directly reduce the viability of the foetus. After the birth of a younger sibling, competition for milk could also occur, as in Galapagos fur seals and sea lions [35], if the older sibling disagrees with the abrupt end of weaning that it experiences. In mandrills, juveniles’ aggressive behaviours toward their infant sibling have never been observed, but older siblings sometimes attempt—and even more rarely succeed—to suckle their mother at the same time as their younger sibling (B.R.T. and M.J.E.C., personal observation). Similar observations were reported from the free-ranging rhesus macaque population in Cayo Santiago, with mothers occasionally nursing two consecutive offspring at the same time [37]. In Cayo Santiago, despite living in a relatively rich environment, short IBIs between two siblings reduced survival chances for both the older and younger sibling, most likely due to the dilution of maternal resources [37]. Recent findings from the same mandrill population showed that the duration of IBIs was highly correlated to female’s rank, with higher-ranking females giving birth to a new offspring every year on average, while lower-ranking females do so once every 2 years [63], a difference that was not observed in provisioned captive mandrills [69]. Therefore, juveniles who entered TTS at the youngest ages might be born to females who can afford the combined energetic costs of gestation and lactation, with little costs for the older sibling, explaining the absence of overt mother–offspring conflict during TTS. Whether mandrill juveniles could pay other costs, similar to those of rhesus macaques, such as decreased survival or slower development for instance, when having a close-in-age younger sibling, remains to be studied.

For young mandrills, the birth of a younger sibling induces an abrupt decrease in maternal care, terminating the weaning process for those who enter TTS at a young age. Juveniles’ reactions to TTS appeared to be sex-specific, with females tending to increase affiliation with their mother while males decreased their association with the mother, but both sexes did not show more conflicts with the mother or more anxiety-related behaviours. These results suggest that, although TTS could induce mother–offspring conflict and sibling competition over maternal investment, young mandrills adjust their developmental pace to their mother’s reproductive pace. Overall, our study adds to recent studies in primates investigating this intriguing developmental milestone, and highlights a variety of reactions across species, probably mirroring contrasts in life histories and ecologies.

## Data Availability

All datasets and codes used in this study are available at Dryad [95]. Supplementary material is available online [96].
